# Malawian children with uncomplicated and cerebral malaria have decreased activated Vγ9Vδ2 γδ T cells which increase in convalescence

**DOI:** 10.1371/journal.pone.0223410

**Published:** 2019-10-10

**Authors:** Visopo Harawa, Madi Njie, Thomas Keller, Kami Kim, Anthony Jaworowski, Karl Seydel, Stephen J. Rogerson, Wilson Mandala

**Affiliations:** 1 Biomedical Sciences Department, College of Medicine, University of Malawi, Blantyre, Malawi; 2 Malawi-Liverpool Wellcome Trust Clinical Research Programme, Blantyre, Malawi; 3 Blantyre Malaria Project, Blantyre, Malawi; 4 Department of Medicine at the Doherty Institute, University of Melbourne, Melbourne, Australia; 5 University of South Florida, Tampa, Florida, United States of America; 6 Department of Infectious Diseases, Monash University, Melbourne, Australia; 7 School of Health and Biomedical Sciences, RMIT University, Melbourne, Australia; 8 Michigan State University, East Lansing, Michigan, United States of America; 9 Academy of Medical Sciences, Malawi University of Science and Technology, Thyolo, Malawi; Instituto Rene Rachou, BRAZIL

## Abstract

Malaria is responsible for almost half a million deaths annually. The role of Vγ9Vδ2 γδ T cells in malaria is still unclear. Studies have reported an association between this cell subset and malaria symptoms and severity. Profiles of Vγ9Vδ2 γδ T cells in bigger cohorts with different levels of clinical severity have not been described. Proportion, numbers, and activation status of Vγ9Vδ2 γδ T cells were measured by flow cytometry in 59 healthy controls (HCs), 58 children with uncomplicated malaria (UM) and 67 with cerebral malaria (CM,) during acute malaria and in convalescence 28 days later. Vγ9Vδ2 γδ T cell were lower in children presenting with UM and CM than in HCs. Cell counts did not vary with malaria severity (CM median counts 40 x 10^3^ cells/μL, IQR [23–103]; UM median counts 30 x 10^3^ cells/μL [10–90], P = 0.224). Vγ9Vδ2 γδ T cell counts increased during convalescence for UM (70 [40–60] x 10^3^ cells/μL and CM (90 [60–140] x 10^3^ cells/μL), to levels similar to those in HCs (70 [50–140] x 10^3^ cells/μL), p = 0.70 and p = 0.40 respectively. Expression of the activation markers CD69 and HLA-DR on Vγ9Vδ2 γδ T cells was higher in malaria cases than in controls (HCs vs UM or CM, p < 0.0001) but was similar between UM and CM. HLA-DR expression remained elevated at 28 days, suggesting sustained activation of Vγ9Vδ2 γδ T cells during recovery. Vγ9Vδ2 γδ T cell proportions and cells counts were suppressed in acute disease and normalized in convalescence, a phenomenon previously hypothesized to be due to transient migration of the cells to secondary lymphoid tissue. The presence of highly activated Vγ9Vδ2 γδ T cells suggests that this T cell subset plays a specific role in response to malaria infection.

## Introduction

Malaria causes over 400,000 deaths every year; with > 70% of these deaths occurring in children less than 5 years old [1]. The host immune response to malaria can be protective or pathological. Vγ9Vδ2 γδ T cells make up 80% of γδ T cells in peripheral blood and have been associated with malaria symptoms and severity [2]. In general, γδ T cells act as a bridge between the innate and adaptive immune response [3,4]. They express both innate and adaptive immune characteristics that are similar to those of T cells, NK cells and antigen presenting cells [5].

γδ T cells exhibit innate characteristics as they respond quickly to foreign antigens without the need for major histocompatibility complex presentation, exhibit limited TCR diversity and are rapidly stimulated in early phases of immune responses [3,5,6]. During malaria Vγ9Vδ2 γδ T cells are activated by malaria phosphoantigens [4,7].

Characteristics of the adaptive immune response displayed by Vγ9Vδ2 γδ T cells include a memory phenotype, possession of a junctionally-diverse T cell receptor (TCR), and the ability to undergo either anergy or expansion depending on the availability of co-stimulation [5]. Their effector functions include direct cytotoxicity to pathogens or infected cells as well as the production of cytokines [3,8–10]. Vγ9Vδ2 γδ T cells have been associated with malaria symptoms and severity [2] and a decrease in numbers of these cells in peripheral blood is thought to result in tolerance to clinical malaria and reduced disease severity [2]. Some studies have reported that during primary malaria infection there is an expansion of the Vγ9Vδ2 subset in malaria naive humans, a phenomenon that is different from that observed in malaria-exposed individuals [6,11–14]. Vγ9Vδ2 γδ T cells were also observed to expand preferentially compared to αβ T cells when PBMC from malaria-naive and malaria-immune subjects were incubated with *P*. *falciparum* infected red blood cells [8,9]. The response of γδ T cells to stimulation *in vitro* with malaria antigens is characterised by proliferation as well as production of cytokines including IFN-γ, IL-1β, and TNF-α which have been associated with both malaria protection and pathology [8,11].

In animal models of malaria, γδ T cell deficient mice were observed to have higher liver parasite burdens than control mice, suggesting that γδ T cells could play a role in controlling liver stage parasitemia [12,15]. γδ T cells exert anti-parasitic functions through production of cytotoxic mediators including perforin and granzymes which may target infected hepatocytes [16]. In mouse experimental CM (ECM) models, γδ T cells accumulated in the brains of mice that developed ECM after IL-2 treatment. Administration of anti- γδ T cell antibodies delayed the onset of the ECM from day 6 to day 18 after infection [17]. These findings indicate that γδ T cells could have a role in the pathogenesis of ECM.

The role of Vγ9Vδ2 γδ T cells in human clinical malaria infection is still not clear [15]. Some studies have reported an increase in numbers and activation of these cells suggesting that they might contribute to pathology of malaria [8,9]. Most of these studies were performed in naive adults rather than children from malaria endemic areas [18]. Furthermore, profiles of Vγ9Vδ2 γδ T cells in different clinical malaria groups have not yet been described. Therefore, we hypothesized that children with cerebral malaria (CM) have higher levels of activated Vγ9Vδ2 γδ T cells than children with uncomplicated malaria (UM) and healthy controls without malaria (HCs).

## Materials and methods

### Study area and study population

A total of 184 children aged between 6 and 144 months were studied. Children presenting with UM (n = 58) and CM (n = 67) were recruited at Queen Elizabeth Central Hospital (QECH) and HCs (n = 59) from Ndirande Health Centre (NHC) in Blantyre, Malawi from January 2016 to June 2017. The two health facilities are within Blantyre urban area and are not very far from each other. For HCs we targeted a hospital that was offering expanded program on immunization services. There are no known differences in malaria transmission patterns between the two health facilities from where the children were enrolled and some of the children enrolled at the QECH (presenting with either UM or CM) actually came from Ndirande area where healthy controls were recruited from. HCs were well children who had no parasites detected on thick blood film examination and had no other known infection at the time of recruitment [19]. UM cases were defined as children with fever who had malaria parasites detected on thick blood film but did not have severe malaria symptoms or signs [19]. CM was defined based on WHO definition of fever (temperature greater than 37.5°C) with asexual stage *P*. *falciparum* parasites on blood film microscopy combined with a Blantyre coma score (BCS) of 2 or less at admission and 4 hours later, after ruling out other potential causes of coma, such as seizures or hypoglycemia [20]. HIV-infected participants were excluded from the final statistical analysis as it is well known that HIV infection independently alters the host immune response to malaria [21,22]. Informed consent was obtained from parents or guardians of all the children enrolled in the study. This study was approved by the University of Malawi College of Medicine Research and Ethics Committee (COMREC). A 5-mL venous blood sample was collected in sodium heparin tubes from each participant at recruitment and from children with UM or CM at a hospital visit 28 days post treatment (in convalescence). CM participants were treated with IV artesunate while UM participants were treated with lumefantrine artesunate which is in line with the current national guidelines for treating these two clinical types of malaria [19].

### Gamma Delta T Cell Phenotyping

A volume of 50 μL of blood was stained with 0.5 μL of anti-CD3-APC (BD Biosciences Cat: 327364), 1.5 μL anti-Vγ9Vδ2 γδ -FITC (BD Biosciences Cat: 337364), 5 μL anti-HLA-DR PerCP (BD Biosciences Cat: 347364) and 5 μL anti-CD69-PE (BD Biosciences Cat: 555531). Samples were incubated for 30 minutes in the dark at 4°C. Two mL of 1x FACS lysing solution was added to each tube and incubated in the dark for 10 minutes at room temperature. Cells were washed twice with 2 mL of FACS wash buffer. Cells were fixed with 200 μL of PBS / 4% formaldehyde. Sample tubes were kept in the dark at 4°C if not acquired immediately. Samples were acquired on CyAn ADP flow cytometer (Beckman Coulter, CA. USA) and analysed using Flowjo software version 10.1 (Tree Star Inc, OR. USA). Refer to S1 Fig for the gating strategy.

### pHRP2 determination

pHRP2 was measured using ELISA. Plasma was diluted at a ratio of either 1:200 or 1:500 in phosphate-buffered saline. These samples, as well as a titration of a stock of recombinant pHRP2, were plated in duplicate (100μL/well) onto a plate precoated with anti- pHRP2 antibody (Cellabs). The manufacturer’s protocol was followed except that all incubations were carried out at 37°C in a humidified chamber instead of at room temperature. Samples were incubated for 1-hour followed with ex- tensive washing with PBS/0.1% Tween, after which 100 μL of conjugated antibody were plated and allowed to incubate for 1 hour. The conjugate was subsequently washed off and 100 μL of substrate were added for 15 minutes, during which color change was observed. This reaction was stopped with 50 μL of stop solution, and the plate was analyzed at an optical density (OD) of 450. A standard curve was generated from the recombinant protein, and pHRP2 concentrations in the samples were calculated using the standard curve.

### Statistical analysis

**Fig 1:**
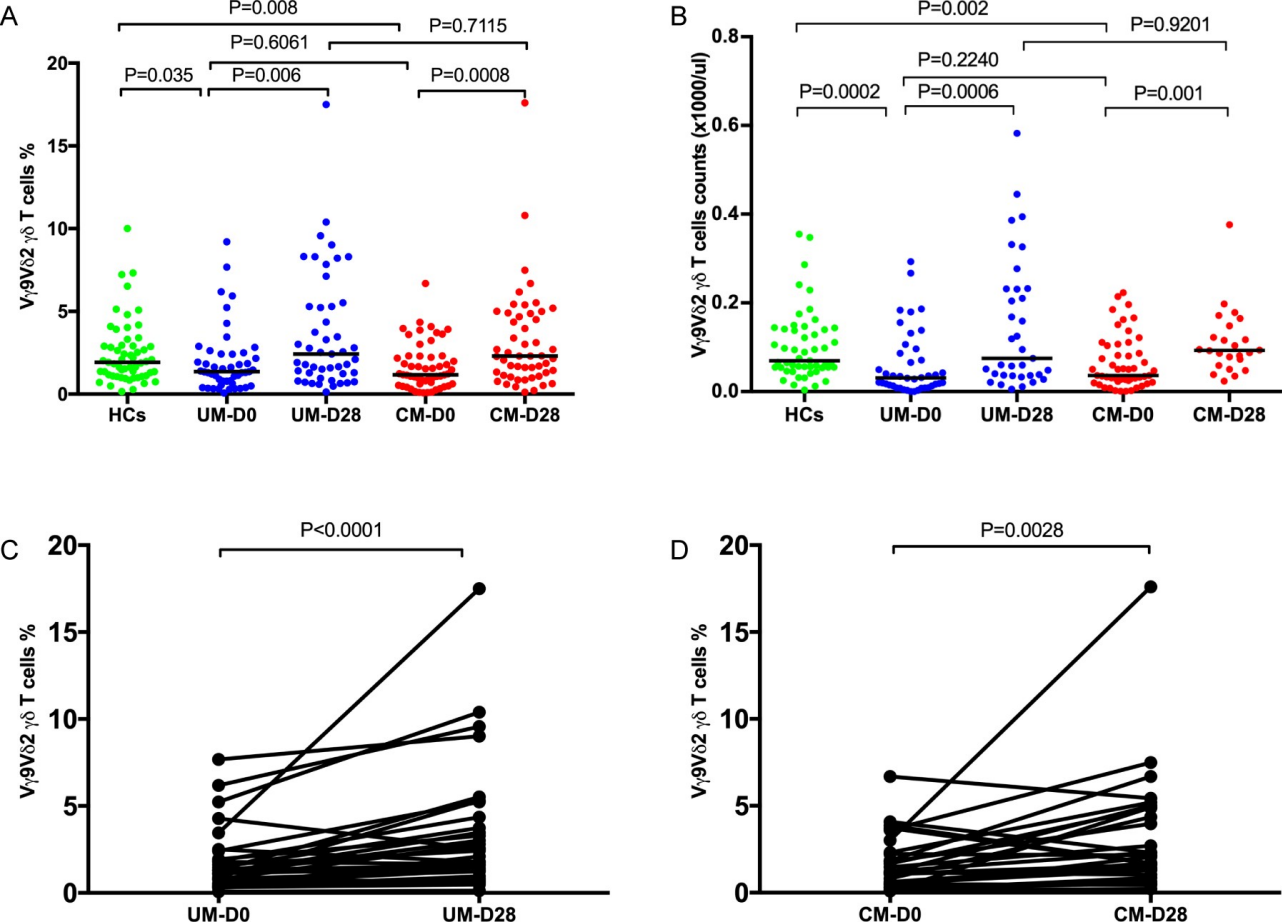
Comparison of peripheral blood Vγ9Vδ2 γδ T cells in healthy controls and malaria children. (A) Median proportions and (B) absolute numbers in children presenting with uncomplicated malaria (UM, n = 58), cerebral malaria (CM, n = 67) and healthy controls (HC, n = 59) at hospital presentation (D0) and in convalescence (D28). (C) Paired analysis of UM at D0 and D28 showing changes in Vγ9Vδ2 γδ T cell within the group. (D) Paired analysis of CM at D0 and D28 showing changes in Vγ9Vδ2 γδ T cell within the group. Bar indicates median value. Mann Whitney U test was used to compare medians of paired groups and p value of 0.05 was considered statistically significant.

**Fig 2:**
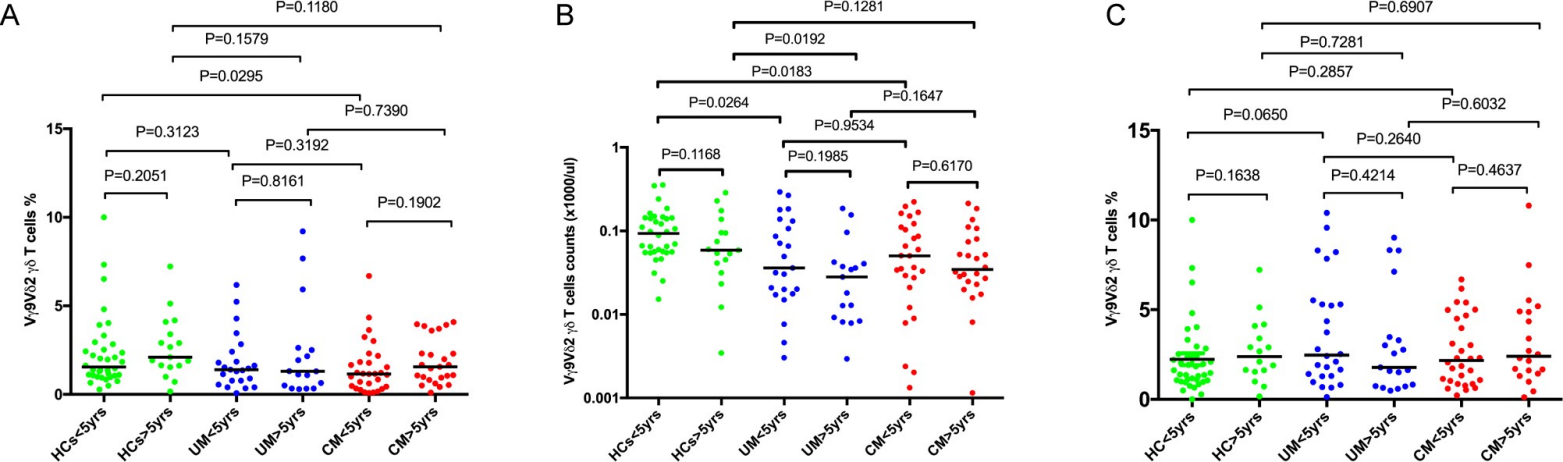
Comparison of medians of peripheral blood Vγ9Vδ2 γδ T cells within each group stratified by age. (A) (% of all T cells) and absolute numbers (B) (HCs < 5yrs n = 34; HCs ≥5yrs n = 17; UM < 5yrs n = 23; UM ≥5yrs n = 17 and CM < 5yrs n = 27; CM ≥5yrs n = 24) at hospital presentation. (C) Comparison of median proportions of peripheral blood Vγ9Vδ2 γδ T cells in convalescence (28 days post treatment) between cases and controls. Bar indicates median value. Mann Whitney U test was used to compare medians of paired groups and p value of 0.05 was considered statistically significant.

**Fig 3:**
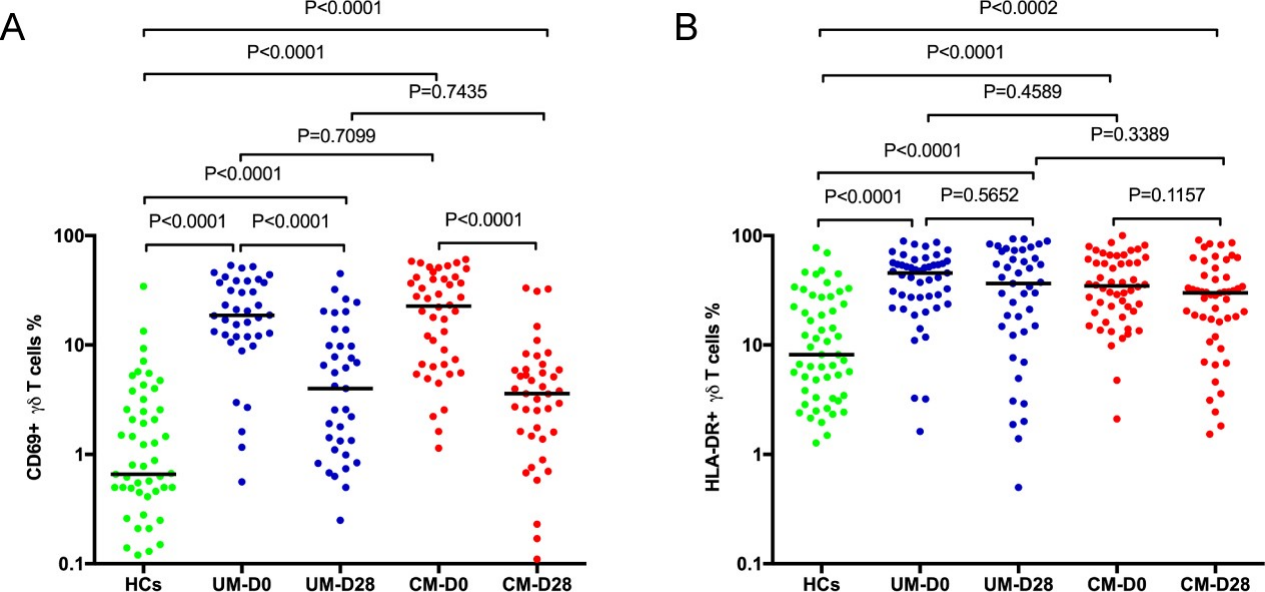
Comparison of the proportions of Vγ9Vδ2 γδ T cells expressing activation markers (A) CD69+ Vγ9Vδ2+ γδ T cells (B) HLA-DR+ Vγ9Vδ2+ γδ T cells in children presenting with uncomplicated malaria (UM, n = 50), cerebral malaria (CM, n = 56)) and healthy controls (HC, n = 57) at hospital presentation (D0) and in convalescence (D28) for cases. Bar indicates median value. Mann Whitney U test was used to compare medians of paired groups and p value of 0.05 was considered statistically significant.

Data were analysed using GraphPad Prism 5 (GraphPad, CA, USA), R package 3.5.2 and Xgboost. Medians and inter quartile ranges (IQR) were computed for continuous variables after log transformation. Kruskal-Wallis test was used to compare medians across more than two groups and between-group comparisons were assessed with Dunn’s multiple comparison test. Correlations between age and Vγ9Vδ2 γδ T cells presented in S2 Fig were plotted using R. Graphs in Figs 1, 2 and 3 were plotted using GraphPad Prism 5. Differences in medians were considered to be statistically significant if the p values were less than or equal to 0.05. We used a machine learning model to predict the clinical groups (health controls or uncomplicated malaria or cerebral malaria); gradient boosted trees were fitted using infection as the predictor variable with Xgboost in the R programming language [23]. To determine the accuracy of the model in predicting clinical groups a confusion matrix (error matrix) was constructed which summarises correct and incorrect predictions. In order to use all of the information that was available, missing data were imputed using bagged trees [24]. All pre-preprocessing of the data was performed with the R packages recipes. The data were centered and scaled before prediction was performed. An initial training and validation split of 2/3 and 1/3 was done, followed by Bayesian optimization over the following parameters: eta (.1-.8), max-depth (3–6), subsample (.5–1.0), lambda (1, 5), alpha (0,5) by searching over a 10-fold split of the training data with the package rBayesianOptimization.

## Results

### Characteristics of study participants

A total of 184 children (CM = 67, UM = 58, HCs = 59) aged between 6 and 144 months were recruited in this study. Children with malaria were older (median age 48 months for UM and 42 months for CM vs 24 months for HCs) and had lower peripheral blood levels of lymphocytes (median cell counts x 10^3^ /μL for UM 2.0 and CM 3.0 vs HCs 5.0) and hemoglobin (median concentration for UM 10.0 g/dL and CM 8.0 g/dL vs HCs 12.0 g/dL) compared to HCs (Table 1).

**Table 1:** Characteristics of study participants.

	Clinical groups			
	Healthy controls	Uncomplicated malaria	Cerebral malaria	P value
Sample size (n) for each group	59	58	67	
Number (%) of male subjects	16 (27.1)	35 (60.3)	34 (50.7)	
Median age (months)	24 (12–66)	48 (24–84)	42 (24–72)	0.0018
Median parasite /HRP2 load (ng/mL)	NA	74.2 (22.6–362.0)	618.0 (236.0–2020.0)	<0.0001
White blood cells (x 10^3^/μL)	8.0 (6.4–9.9)	7.0 (5.9–10.2)	9.0 (6.1–12.3)	ns
Haemoglobin (g/dL)	12.0 (11.0–12.8)	10.0 (8.6–11.6)	8.0 (6.9–9.3)	<0.0001
Lymphocytes (%)	57.0 (49.7–63.7)	29.0 (18.2–43.7)	38.0 (26.1–46.4)	<0.0001
Lymphocytes (x 10^3^/μL)	5.0 (3.6–5.4)	2.0 (1.5–3.1)	3.0 (2.1–4.7)	<0.0001

Results are presented as medians and interquartile ranges (25–75%) except for sex which is presented as an absolute number and a percentage. NA means not applicable. Kruskal Wallis test was used for group analysis, p value of ≤ 0.05 was considered statistically significant

### Peripheral blood Vγ9Vδ2 γδ T cell proportions and counts decreased during acute malaria and normalized in convalescence

The median proportions of Vγ9Vδ2 γδ T cells were lower in children with UM and CM than in controls at hospital presentation (Fig 1A; UM median proportion 1.36%, [0.68–2.43] vs HCs (1.92% [1.11–2.93] p = 0.035; CM median proportion 1.20% IQR [0.62–2.13] vs HCs (1.92% [1.11–2.93] p = 0.008). Similarly, the absolute numbers of Vγ9V2 γδ T cells were lower in children with UM and CM than in controls at hospital presentation (Fig 1B: median counts for UM (30 x 10 ^3^ cells/μL, IQR [10–90] vs HCs (70 x 10 ^3^ cells/μL [50–140] p = 0.0002; CM median counts (40 x 10^3^ cells/μL, IQR [23–103] vs HCs (70 x 10 ^3^ cells/μL [50–140] p = 0.002). There was no difference in median proportions between children who had UM (1.36%, [0.68–2.43]) and those who had CM (1.20% IQR [0.62–2.13], P = 0.662) and median counts for CM (40 cells/μL, IQR [23–103] and UM (30 cells/μL, [10–90], P = 0.224 (Fig 1A and 1B).

Both proportions and counts of Vγ9Vδ2 γδ T cells in UM and CM cases increased to similar levels observed in HCs. The median proportions of Vγ9Vδ2 γδ T cells in convalescence; UM (2.42% [1.24–5.30], p = 0.261, and CM (2.30% [1.14–4.87], p = 0.299) were similar to those HCs (1.92% [1.11–2.93]. There was also no significant difference between UMs and CMs (p = 0.712) Fig 1A. The median counts of Vγ9Vδ2 γδ T cells in convalescence; UM (70 cells/μL [40–60]) and CM (90 cells/μL [60–140]) were similar to those HCs (70 cells/μL [50–140]) (p = 0.70 and p = 0.40 respectively). There was also no significant difference between UM and CM (p = 0.920) Fig 1B. Proportions of Vγ9Vδ2 γδ T cells were lower during acute infection (D0) than during convalescence (D28) for both UM and CM cases; UM (D0 1.36% [0.675–2.43] vs D28 2.42% [1.24–5.30], P = 0.006), CM (D0 1.20% [0.620–2.31] vs D28 2.30% [1.14–4.87], p = 0.002), Fig 1A, Fig 1C and Fig 1D.

### Vγ9Vδ2 γδ T cells proportions and counts did not vary with age

Because the children with HC, UM, and CM were not age matched, the relationship between peripheral blood Vγ9Vδ2 γδ T cells levels and age was assessed. Levels of Vγ9Vδ2 γδ T cells in children below the age of 5 years and children 5 years old and above were compared. Proportions of Vγ9Vδ2 γδ T cells were similar in children below 5 years and those above 5 years in all the three groups; HCs (<5yrs, 1.6% [1.06–2.75] vs ≥5yrs 2.1% [1.58–3.75]), p = 0.21; UM (<5yrs, 1.4% [0.78–2.41] vs ≥5yrs 1.3% [0.42–2.57], p = 0.82); CM (<5yrs, 1.2% [0.41–1.92] vs ≥5yrs 1.56% [0.88–2.96], p = 0.19). (Fig 2A), (S2 Fig).

Similarly, absolute counts for Vγ9Vδ2 γδ T cells were similar in children below 5 years and those above 5 years in all the three groups; HCs (<5yrs, 90 cells/μL [60–140] vs ≥5yrs 60 (cells/μL) [40–120], p = 0.12), UM (<5yrs, 40 (cells/μL) [20–130] Vs ≥5yrs 30 (cells/μL) [10–40], p = 0.20), CM (<5yrs, 50 (cells/μL) [20–110] Vs ≥5yrs 30 (cells/μL) [20–80], p = 0.62). (Fig 2B). Levels were also similar in convalescence (Fig 2C).

### Malaria infection was associated with activated Vγ9Vδ2 γδ T cells

Compared to HCs, expression of both CD69 and HLA-DR on Vγ9Vδ2 γδ T cells was higher in malaria cases during acute disease (Fig 3A and Fig 3B). The expression of CD69 and HLA-DR did not vary between UM and CM, (Fig 3A and Fig 3B). Within clinical malaria groups, the proportion of Vγ9Vδ2 γδ T cells expressing CD69 significantly decreased during convalescence, but this was not observed for their expression of HLA-DR (Fig 3A and Fig 3B).

### Machine learning outcome prediction and feature categorization

The prior sections considered the effect of Vγ9Vδ2 γδ T cells in malaria with one variable at a time, variables such as case type, acute disease or convalescence, and age. We therefore employed a machine learning model, boosted trees with XGBoost, to help deal with the potentially complex relationships of the data when considering all of the variables together in a predictive model. Using a combined model allows us to compare the relative weight or importance of a variable.

**Fig 4:**
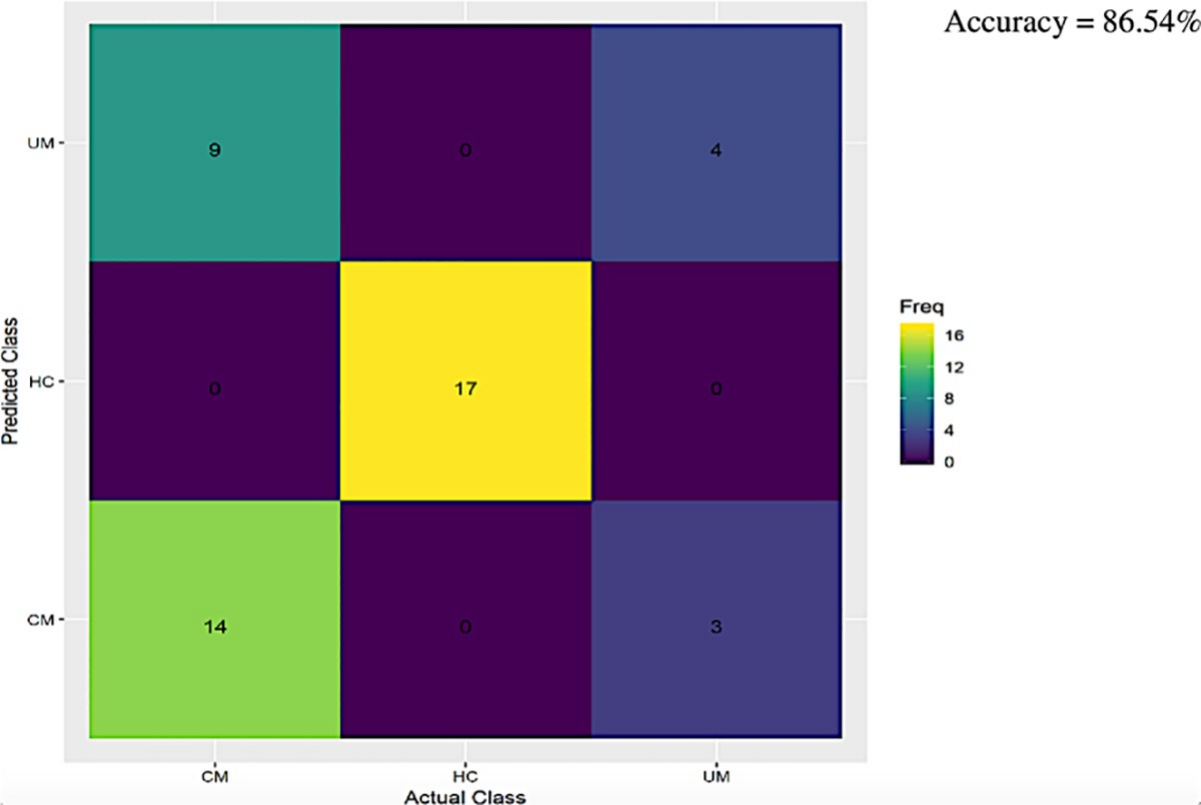
Machine learning outcome predicting infection status of plasmodium falciparum in clinical groups. The numbers in each class represent the number of correctly predicted cases from the trained Xgboost model for each category. Accuracy refers to the fraction of correct predictions from the trained model. CMs are mostly classified as CM, however there is some misclassification both in UM (as CM) and CMs misclassified as UM. HCs are consistently classified correctly.

We used XGBoost to determine whether we could predict malaria clinical categories using the immunological and clinical variables collected for each clinical group. To further investigate the relationship between Vγ9Vδ2 γδ T cells count, activation markers (CD69 & HLA-DR) and age with the malaria infection groups examined here, a confusion matrix was developed (Fig 4). The model accurately predicted 95% HCs and 76% CMs, but only 49% UMs. It was successful in discriminating uninfected children (HCs) from children with malaria (UM and CM). The model performed less well discriminating between UM and CM, particularly in children presenting with UM. The positive predictive values for each clinical group were as follows: HCs = 0.82; UM = 0.57 and CM = 0.73. The overall accuracy (e.g. the fraction of correct predictions on the dataset) in predicting whether someone was a CM or UM or HC was 86.54% (P = 1.279E-13) and a kappa p value of 0.79 suggest that its prediction levels were good (Fig 4). The model was very good at discriminating HCs from UMs and CMs, and generally predicted CM well. The majority of misclassifications occurred for UMs (both UMs misclassified as CMs, and CMs misclassified as UM). Biologically, at least for variables used to build this model, there is considerable overlap between CM and UM, consistent with the prior bivariate results.

**Fig 5:**
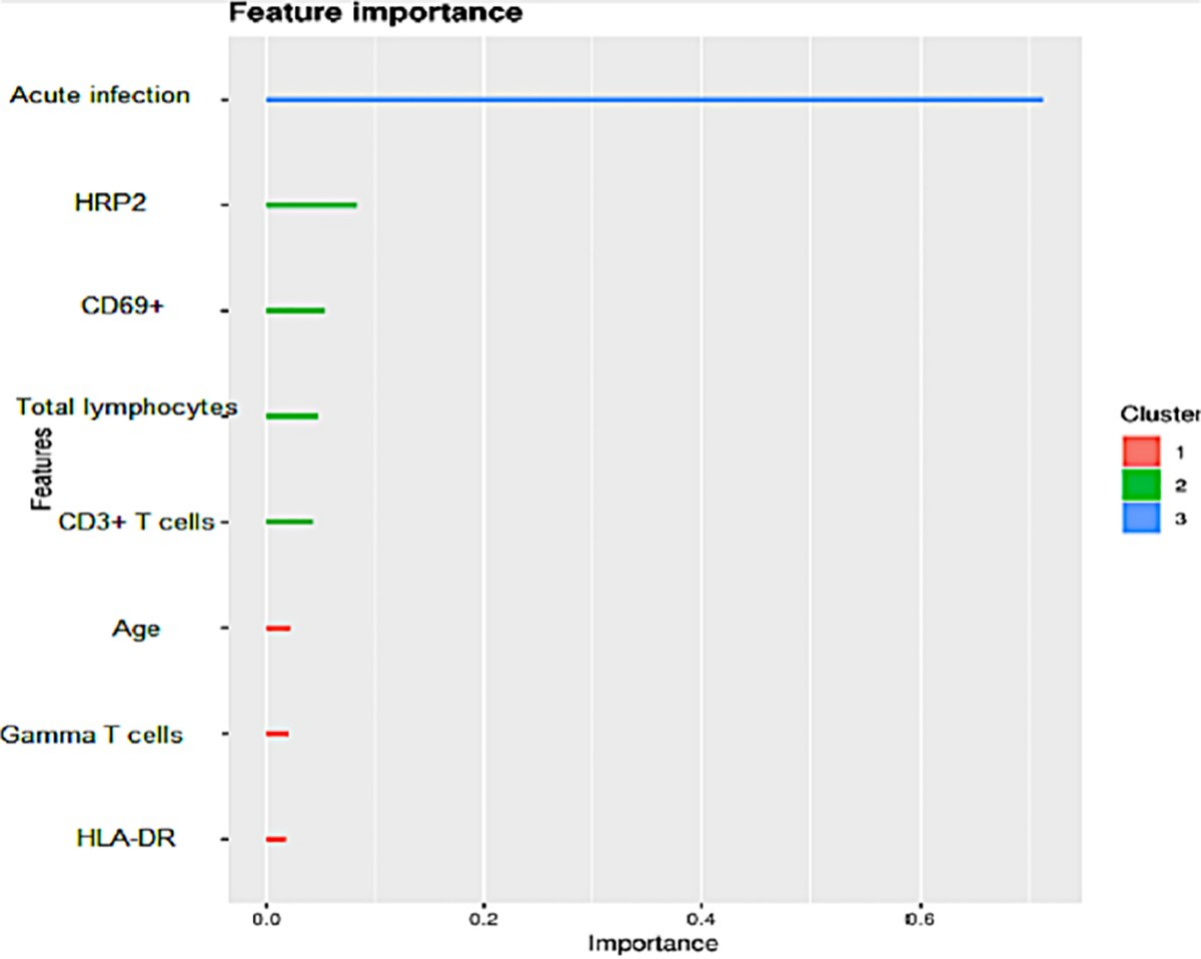
Categorization of features in clinical groups based on their importance in prediction of infection status of study participants. Importance refers to the relative information gain in the decision tree model when the variable is included. The colours refer to a 1-d clustering or grouping that was performed, such that variables with similar importance were clustered together (essentially a high, middle, and low importance cluster).

The model was also used to predict which feature (Vγ9Vδ2 γδ T absolute cell counts, HLA-DR, CD3 T cells, CD69, age, acute infection and convalescence) was most important with regards to prediction of clinical groups. The model classified the features into three clusters (subsets) based on similarity in predicting infection status. Acute infection was shown to be the highest predictor in the combined dataset for discriminating infected (UM and CM) from uninfected children (HCs); with a gain value of 0.71 followed by HRP2 and CD69+ T cells with gain values of 0.08 and 0.05 respectively (Fig 5). Qualitatively, acute infection was consistently the single most important predictor found in the boosted trees, all other variables added less to the model when included.

## Discussion

To understand the role of Vγ9Vδ2 γδ T cells in *P*. *falciparum* malaria, the number, proportion and activation status were compared in three clinical groups of children (UM, CM and HCs). Both proportions and absolute counts of Vγ9Vδ2 γδ T cells were low in acute disease increasing in convalescence 28 days later. The expression of activation markers CD69 and HLA-DR was higher in acute cases than in controls, as previously shown by others [23], and HLA-DR expression remained elevated in convalescence above levels observed in HCs. Notably, during acute disease, neither proportions of Vγ9Vδ2 γδ T cells nor their expression of CD69 and HLA-DR varied with disease severity. Acute infection was the most important feature predictor in the combined dataset for discriminating between the infection groups studied herein.

The decrease in proportions and numbers of Vγ9Vδ2 γδ T cells in the peripheral blood during malaria infection observed in this cohort could suggest that these cells migrate into lymphoid tissues as previously hypothesized in different reports [20,25]. CD69 has been shown to be important in migration of lymphocytes [18,19]. The findings of our study are in agreement with a cross-sectional study performed in The Gambia that investigated γδ T cell profiles in children who presented with CM (n = 7), severe malaria anaemia (n = 5) and UM (n = 7), and found a decrease in frequencies of γδ T cells in peripheral blood in all the patient groups at hospital presentation [18].

When T cells are activated in response to their cognate antigen they express activation markers including CD69 and HLA-DR on their surface, which augment the immune response [26,27]. CD69 is a membrane protein which is activated early in infection, usually within 2 hours of T cells stimulation *in vitro* with mitogens [28]. Its expression peaks after 12 hours [29]. This activation results in cell proliferation and secretion of cytokines IL-12 and IFN-γ which play inflammatory immune roles [30]. CD69 expression is crucial in the migration of T cells to bone marrows and their differentiation into memory phenotype [26,27]. Malaria disease has previously been characterized by highly activated lymphocyte subsets that express activation surface markers such as CD69, HLA-DR and CD38 [20]. CD69 and HLA-DR represent early and late activation markers respectively. In this cohort, there were no differences in levels of activation of the Vγ9Vδ2 T-cells between UM and CM groups during acute disease suggesting that the activation status of these cells in peripheral blood was independent of disease severity. This agrees with the findings of Hviid et al. who found no difference in the levels of activation of γδ T cells between UM and CM cases [18].

Machine learning models demonstrated that acute infection was the most important predictive feature in the combined dataset for discriminating infection status (healthy controls from infected). Both UM and CM showed sustained activation of γδ T cells.

Other researchers have hypothesized that expansion of γδ T cells in malaria naïve people and a lack of expansion in clinically immune individuals may implicate γδ T cells in malaria pathogenesis in malaria naïve individuals [13]. The proportions of γδ T cells in peripheral blood correlates with clinical disease intensity in malaria naïve individuals infected with malaria [13]. The differences in the findings between the previous studies and the current study may be explained by the current study using samples from malaria-exposed children while previous studies used samples from malaria naïve adults. It should be noted that most previous studies examined at γδ T cells, in general, while this study examined at a specific subset of γδ T cells (Vγ9Vδ2 γδ T cells) which is the most common in peripheral blood.

This study has several limitations, including that no information is available on the participants’ previous exposure to malaria. Of interest would have been to investigate whether previous exposure had an effect on the proportions of Vγ9Vδ2 γδ T cells in peripheral blood. Previous studies have shown that exposure to malaria is associated with lower proportions of peripheral blood Vγ9Vδ2 γδ T cells [21–22]. Although children in the control group were younger than cases, when groups were stratified based on age (below and above the age of 5 years), there were no differences in the proportions and numbers of Vγ9Vδ2 γδ T cells in HCs, UM and CMs. γδ T cells continued to express activation markers 28 days after malaria infection, so a longitudinal approach with samples collected at more time points over a longer period such as six months would have been informative about the kinetics of V9Vδ2 γδ T cells activation.

Another limitation was that no measure of Vγ9Vδ2 γδ T cells function was performed, which may or may not have correlated with counts and could have provided important insights in the role of these cells in malaria immunity or pathogenesis in children. It would have been interesting to see whether the proportion of activated Vγ9Vδ2 γδ T correlated with the proportion of IFN-γ-producing cells or whether the activated cells showed increased degranulation upon stimulation with parasite antigens. This would have corroborated the presented data and offered further insights into the role of Vγ9Vδ2 γδ T cells in malaria. Furthermore, in addition to characterizing Vγ9Vδ2 γδ T cells, it would have also been informative to do the same with other subsets of γδ T cells like Vδ1 γδ T cells.

## Conclusions

The findings of this study indirectly provides some support to the hypothesis that during malaria infection Vγ9Vδ2 γδ T cells, just like other lymphocyte subsets, transiently migrate into the secondary lymphoid tissue and re-emerge during convalescence [31]. In secondary lymphoid tissues these cells encounter malaria antigens presented by antigen-presenting cells and are consequently activated [31,32]. The presence of highly activated CD69+ cells during acute disease which diminish in convalescence, suggests that the activation occurs in response to the malaria infection. Additionally, the lack of difference in the activation status of the cells between UM and CM suggests that Vγ9Vδ2 γδ T cells activation occurs early in malaria infection and may have a minimal role in pathogenesis of severe malaria.

## Supporting information

S1 Fig Gamma/Delta T gating strategy using whole blood. (A) A forward and side scatter plot gated on lymphocytes. (B) Lymphocytes singlets plot. (C) Vγ9Vδ2+ CD3+ T cells gated on lymphocytes singlets. (D) CD69+ Vγ9Vδ2+ T cells gated on Vγ9Vδ2+ CD3+ T cells. (E) HLA-DR+ Vγ9Vδ2+ T cells gated on Vγ9Vδ2+ CD3+ T cells. (F) Fluorescence minus one control for Vγ9Vδ2+ CD3+ T cells. (G) Fluorescence minus one control for Vγ9Vδ2+ CD69+ T cells. (H) Fluorescence minus one control for Vγ9Vδ2+ HLA-DR+ T cells.
(PDF)

S2 Fig Spearman coefficient analysis to check if there was correlation analysis between ages (years) and Vγ9Vδ2 γδ T cells. Vγ9Vδ2+ γδ T cells absolute counts (cells/μL) in peripheral blood of children stratified by age; (A) Children with uncomplicated malaria, (B) cerebral malaria and (C) healthy controls at hospital presentation.
(TIFF)
