# Supplementary figures and images for: Malawian children with uncomplicated and cerebral malaria have decreased activated Vγ9Vδ2 γδ T cells which increase in convalescence
Source: PLoS One. 2019 Oct 10;14(10):e0223410. doi: 10.1371/journal.pone.0223410 (PMC6786631; doi:10.1371/journal.pone.0223410)

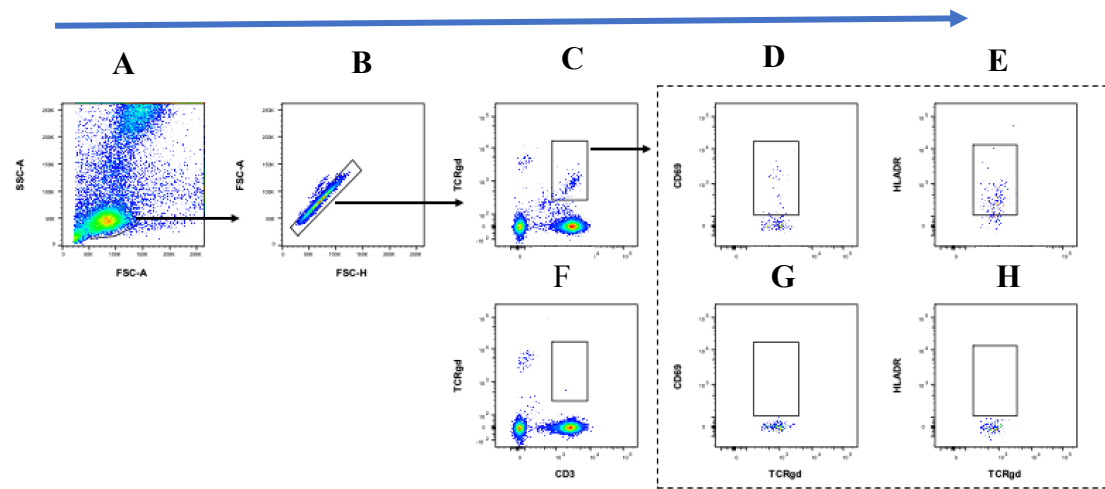

Supplement: S1 Fig — (A) A forward and side scatter plot gated on lymphocytes. (B) Lymphocytes singlets plot. (C) Vγ9Vδ2+ CD3+ T cells gated on lymphocytes singlets. (D) CD69+ Vγ9Vδ2+ T cells gated on Vγ9Vδ2+ CD3+ T cells. (E) HLA-DR+ Vγ9Vδ2+ T cells gated on Vγ9Vδ2+ CD3+ T cells. (F) Fluorescence minus one control for Vγ9Vδ2+ CD3+ T cells. (G) Fluorescence minus one control for Vγ9Vδ2+ CD69+ T cells. (H) Fluorescence minus one control for Vγ9Vδ2+ HLA-DR+ T cells. (PDF) [file pone.0223410.s001.pdf]

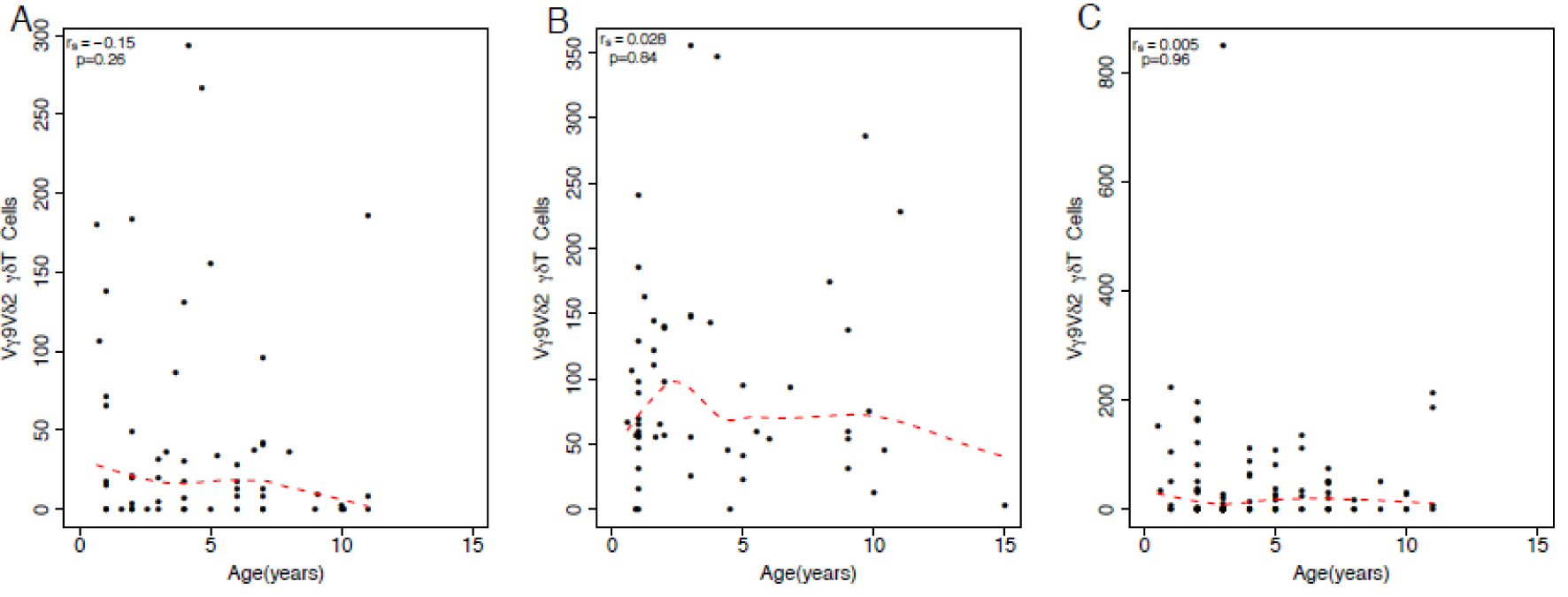

Supplement: S2 Fig — Vγ9Vδ2+ γδ T cells absolute counts (cells/μL) in peripheral blood of children stratified by age; (A) Children with uncomplicated malaria, (B) cerebral malaria and (C) healthy controls at hospital presentation. (TIFF) [file pone.0223410.s002.tiff]
